# A Pathogenic Role for Splenic B1 Cells in SIV Disease Progression in Rhesus Macaques

**DOI:** 10.3389/fimmu.2019.00511

**Published:** 2019-03-19

**Authors:** Gospel Enyindah-Asonye, Anthony Nwankwo, Christopher Hogge, Mohammad Arif Rahman, Sabrina Helmold Hait, Ruth Hunegnaw, Eun-Ju Ko, Tanya Hoang, David J. Venzon, Marjorie Robert-Guroff

**Affiliations:** ^1^Vaccine Branch, National Cancer Institute, National Institutes of Health, Bethesda, MD, United States; ^2^Biostatistics and Data Management Section, National Cancer Institute, National Institutes of Health, Bethesda, MD, United States

**Keywords:** B1 cells, rhesus macaque, simian immunodeficiency virus, exhaustion, T cells

## Abstract

B1 cells spontaneously produce protective natural antibodies which provide the first line of defense against a variety of pathogens. Although these natural antibodies share similar autoreactive features with several HIV-1 broadly neutralizing antibodies, the role of B1 cells in HIV/SIV disease progression is unknown. We report the presence of human-like B1 cells in rhesus macaques. During chronic SIV infection, we found that the frequency of splenic CD11b^+^ B1 cells positively correlated with plasma SIV viral load and exhausted T cells. Mechanistically, we discovered that splenic CD11b^+^ B1 cells express PD-L2 and IL-10, and were able to induce PD-1 upregulation on CD4^+^ T cells *in vitro*. These findings suggest that splenic CD11b^+^ B1 cells may contribute to the regulation of SIV plasma viral load by enhancing T cell exhaustion. Therefore, understanding the mechanisms that govern their function in rhesus macaques may lead to novel therapeutic strategies for impeding HIV/SIV disease progression.

## Introduction

B1 cells are innate-like B cells that spontaneously produce natural antibodies in the absence of infection or immunization (1, 2). These natural antibodies are predominantly of IgM isotype and provide a first line defense against various pathogens. In mice, B1 cells are primarily located in the peritoneal cavity but are also found in the spleen, lymph node (LN), and peripheral blood (1–5). In humans, B1 cells were recently phenotypically characterized as CD3^−^CD19^+^CD43^+^CD27^+^ and reported to be present in peripheral blood and tonsillar tissue (6). The natural antibodies produced by B1 cells tend to be polyreactive and can bind to foreign and self-antigens such as cardiolipin, histones, centromeres and double stranded DNA (1). Thus, B1 cells are involved in maintaining microbial defense and immune homeostasis, and dysfunction of B1 cells is associated with autoimmune diseases. Two broadly reactive human immunodeficiency virus (HIV)-1 antibodies, 2F5 and 4E10 (7–9), display similar polyreactive characteristics suggesting that B1 cells may be a candidate B cell subset from which broadly neutralizing antibodies originate. However, the role of these cells in simian immunodeficiency virus (SIV)/HIV infection is unknown.

Beyond natural antibody secretion and its key role in infection remediation, B1 cell activities affect other elements of the immune system in both stimulatory and suppressive ways. B1 cells strongly induce activation and proliferation of naive T cells across an allogeneic barrier, indicating that B1 cells are efficient antigen presenting cells (10, 11). Among B1 cell subsets, the programmed cell death-ligand (PD-L) 2^+^ subpopulation in mice and the CD11b^+^ subpopulation in humans are particularly efficient at stimulating T cell activation and expansion. B1 cells can also influence T cell differentiation (6, 12, 13). They induce differentiation of naive, CD4^+^ T cells to interleukin (IL)-17–expressing pro-inflammatory T helper (Th) 17 cells, whereas B2 (CD3^−^CD19^+^CD43^−^CD27^−^) cells induce T cell differentiation to regulatory T (T_reg_) cells (11).

B1 cells have been shown to play an immunosuppressive role by spontaneously secreting IL-10, which has been attributed to the CD11b^+^ subpopulation in humans (12). B1 cells constitutively express PD-L1, and a subset (50–70%) of these cells also express PD-L2 (13, 14), key factors responsible for inhibiting T cell receptor (TCR) signaling. PD-L1 and PD-L2 are both ligands for PD-1, the hallmark of exhausted T cells (15, 16). Considering that B1 cells are capable of stimulating T cell expansion, inducing Th17 cell differentiation, and suppressing T cell activity, they would appear to be in a position to heavily influence the nature and direction of T cell immune responses during HIV/SIV infection.

T cell exhaustion, which leads to progressive loss of T cell antigen-specific function, is one of the major hurdles in the efficient treatment of chronic viral infections (17–19). PD-1, an inhibitory surface co-receptor, is a member of the CD28/B7 family that is expressed on T cells, B cells and myeloid-derived cells. Ligation of PD-1 dampens T cell antiviral effector functions, while favoring cell anergy and apoptosis (20, 21). PD-1 over-expression has been associated with T cell dysfunction and elevated viral load in a variety of chronic viral infections such as HIV/SIV and lymphocytic choriomeningitis virus (LCMV) (17, 22, 23). Particularly in the case of HIV/SIV infection, increased PD-1 expression on antigen-specific CD4^+^ and CD8^+^ T cells has been associated with T cell exhaustion and disease progression (16, 17).

Whether B1 cells exist in rhesus macaques and play a role in SIV disease progression is currently unknown. Here, we report the presence of human-like B1 cells in rhesus macaques. Our data suggest that B1 cells residing in the spleen may increase SIV viral load by contributing to T cell exhaustion during the chronic phase of SIV infection. Thus, our data point to a potential contributory role for splenic B1 cells in SIV disease progression.

## Materials and Methods

### Study Animals

Rhesus macaques were maintained at Advanced Bioscience Laboratories, Inc. (Rockville, MD) and at the National Cancer Institute animal facility (Bethesda, MD) under the guidelines of the Association for the Assessment and Accreditation of Laboratory Animal Care and according to the recommendations of the Guide for the Care and Use of Laboratory Animals. Protocols and procedures were approved by the Institutional Animal Care and Use Committee of the respective facility. Bone marrow, inguinal LN and blood samples were collected from a random subset of naïve macaques, acute (2 wk after SIV_mac251_ infection) and chronically infected macaques (40–50 wk after SIV_mac251_ infection). Spleen samples were collected from chronically infected macaques at necropsy. These macaques were previously vaccinated with replicating adenovirus–SIV recombinants and boosted with either ALVAC–SIV recombinant plus SIV gp120 protein or SIV DNA plasmid plus gp120 protein (24). All macaques were 3–4 years old at study initiation and were 4–5 years old at the time of SIV infection. Three groups of macaques were investigated in this cross-sectional study: uninfected/pre-immmunization (*n* = 15), acutely infected (*n* = 14), and chronically infected (*n* = 36). Only 1 macaque had LN samples assessed longitudinally at all three time points. There were 5, 11, and 19 females in the uninfected, acutely-infected and chronically-infected groups, respectively, and 10, 3, and 17 males, respectively, in the same groups. All macaques were screened for Mamu-A^*^01, Mamu-B^*^08, and Mamu-B^*^17 MHC haplotypes. Three, four, and five Mamu-A^*^01 macaques were in the uninfected, acutely-infected and chronically-infected groups, respectively, and 1, 1, and 4 Mamu-B^*^17 macaques, respectively were in the same groups. One Mamu-B^*^08 macaque was in the chronically infected group. No macaque had more than one of these three haplotypes.

### Sample Collection and Preparation

Spleen, inguinal LN and bone marrow single-cell suspensions were prepared by gentle dissection and passed through a 40-μm cell strainer after lysis of RBCs. The cells were washed and resuspended in R10 complete media (RPMI 1640 containing 10% FBS, 2 mM L-glutamine, 1% nonessential amino acids, 1% sodium pyruvate, and antibiotics) (25–27). Rectal pinches were digested with collagenase (2 mg/ml, Sigma Aldrich) for 45 min. Single-cell suspensions were prepared by gentle mincing and filtering through a 40-μm cell strainer (27, 28). The cells were washed and resuspended in R10 complete media. Peritoneal cells were isolated by lavaging the peritoneal cavity with 150 ml PBS and filtering the lavage through a 40 μm cell strainer (5). Peritoneal and rectal cells were used fresh for flow cytometric analysis.

### Flow Cytometric Acquisition

For flow cytometric acquisition, thawed single-cell suspensions were stained on ice for 30 min using manufacturers' suggested optimal concentrations of monoclonal antibodies (mAbs) in the dark. After 30 min, the cells were washed with PBS and resuspended in FACS buffer. At least 500,000 singlet events were acquired on a SORP LSR II (BD Biosciences) and analyzed using FlowJo software (FlowJo, Ashland, OR). For all samples, gating was established using a combination of isotype and fluorescence-minus-one controls.

### Antibodies

The mAbs used in this study are as follows: anti-CD6 (MT-605), anti-CD4 (L200), anti-CD8 (RPA-T8), anti-CD3 (SP34.2), anti-CD20 (2H7), and anti-LAG-3 (T47-530) were obtained from BD Bioscience (San Jose, CA). Anti-PD-L1 (29E.2A3), anti-PD-L2 (24F.10C12), and anti-PD-1 (EH12.2H7) were obtained from Biolegend (San Diego, CA). Anti-CD11b (ICRF44) antibody was obtained from eBioscience (San Diego, CA). Anti-CD19 (J3-119) was obtained from Beckman Coulter (Brea, CA). Anti-CD43 (4-29-5-10-21) and anti-CD27 (0323) were obtained from Invitrogen (Carlsbad, CA). Mouse monoclonal anti-monkey IgM was obtained from Life Diagnostic catalog # 2C11-1-5, (West Chester, PA). Monkey IgM whole molecule was obtained from Rockland (Limerick, PA). Goat anti-monkey IgM-HRP was obtained from Novus (Littleton, CO). Goat anti-monkey IgG (catalog # 70023) and goat anti-monkey IgG-HRP were obtained from Alpha Diagnostic International (San Antonio, TX). Purified rhesus IgG was obtained from the NHP reagent resource.

### Flow Cytometric Detection of IL-10

IL-10 staining was performed by culturing splenocytes from chronically SIV-infected macaques in complete media in the presence of BD Golgistop (1 μl; BD) containing monensin for 4 h prior to cell surface staining. Following surface staining, the cells were fixed and permeabilized using eBioscience intracellular fixation and permeabilization buffer according to the manufacturer's instructions prior to staining with anti-IL-10 (JES3-9D7, eBioscience). Isotype-matched mAb served as negative control for IL-10 staining to demonstrate specificity and to establish background IL-10 staining levels.

### Cell Sorting, Co-culture, and ELISA

Spleen cells from chronically infected animals were stained with anti-CD4, anti-CD3, anti-CD20, anti-CD43, anti-CD27, and anti-CD11b. Aqua Live/Dead viability dye was used to exclude dead cells. After staining, cells were washed, passed through a 40-μm cell strainer, and sorted on an Astrios EQ flow cytometer. Three groups of live cells were sorted (CD3^−^CD20^+^CD43^+^CD27^+^CD11b^+^, CD3^−^CD20^+^CD43^+^CD11b^−^, and CD3^+^CD4^+^) with purity of >85%. CD11b^+^ or CD11b^−^ B1 cells were co-cultured with CD3^+^CD4^+^ T cells in complete media at a 1:3 ratio with sort-purified B1 cells (50,000) and CD3^+^ CD4^+^ T cells (150,000) for 3 days and PD-1 expression was analyzed on CD3^+^ CD4^+^ T cells by flow cytometry.

PBMC from naïve macaques were stained with anti-CD3, anti-CD19, anti-CD20, anti-CD43, and anti-CD27. Aqua Live/Dead viability dye was used to exclude dead cells. After staining, cells were washed, passed through a 40-μm cell strainer, and sorted on an Astrios EQ flow cytometer. Two groups of live cells were sorted (CD3^−^CD19^+^CD43^+^CD27^+^ and CD3^−^CD19^+^CD43^−^CD27^−^) with purity of >85%. These sorted cells were cultured in complete media for 3 days at a concentration of 100,000 cells and the supernatants were evaluated for IgM and IgG secretion. For IgM secretion, ELISA plates were coated with mouse monoclonal anti-monkey IgM. Bound antibodies were detected using anti-IgM monkey HRP. The ELISA color reaction was initiated using TMB substrate (Thermo Scientific, MA). 2M H_2_SO_4_ was used to stop the TMB reaction, and absorbance at 450 nm was measured. The concentrations were determined with a standard curve derived from monkey IgM whole molecule. For IgG secretion, ELISA plates were coated with goat anti-monkey IgG. Bound IgG antibodies were detected using anti-IgG monkey HRP. The ELISA color reaction was conducted as above. The concentrations were determined with a standard curve derived from purified rhesus IgG obtained from the NHP reagent resource.

### Statistical Analysis

For multiple group analyses, we performed the Kruskal–Wallis and Dunn's pairwise multiple comparison tests. For two group comparisons, we performed the nonparametric Mann–Whitney test. Correlation analyses were assessed using the nonparametric Spearman test. The Wilcoxon signed rank test was performed for the coculture assay. All tests were two-tailed and done at the 0.05 alpha level. GraphPad Prism was used for statistical analysis.

## Results

### Human-Like B1 Cells Are Present in the Peripheral Blood and Lymphoid Tissues of Rhesus Macaques

**Figure 1:**
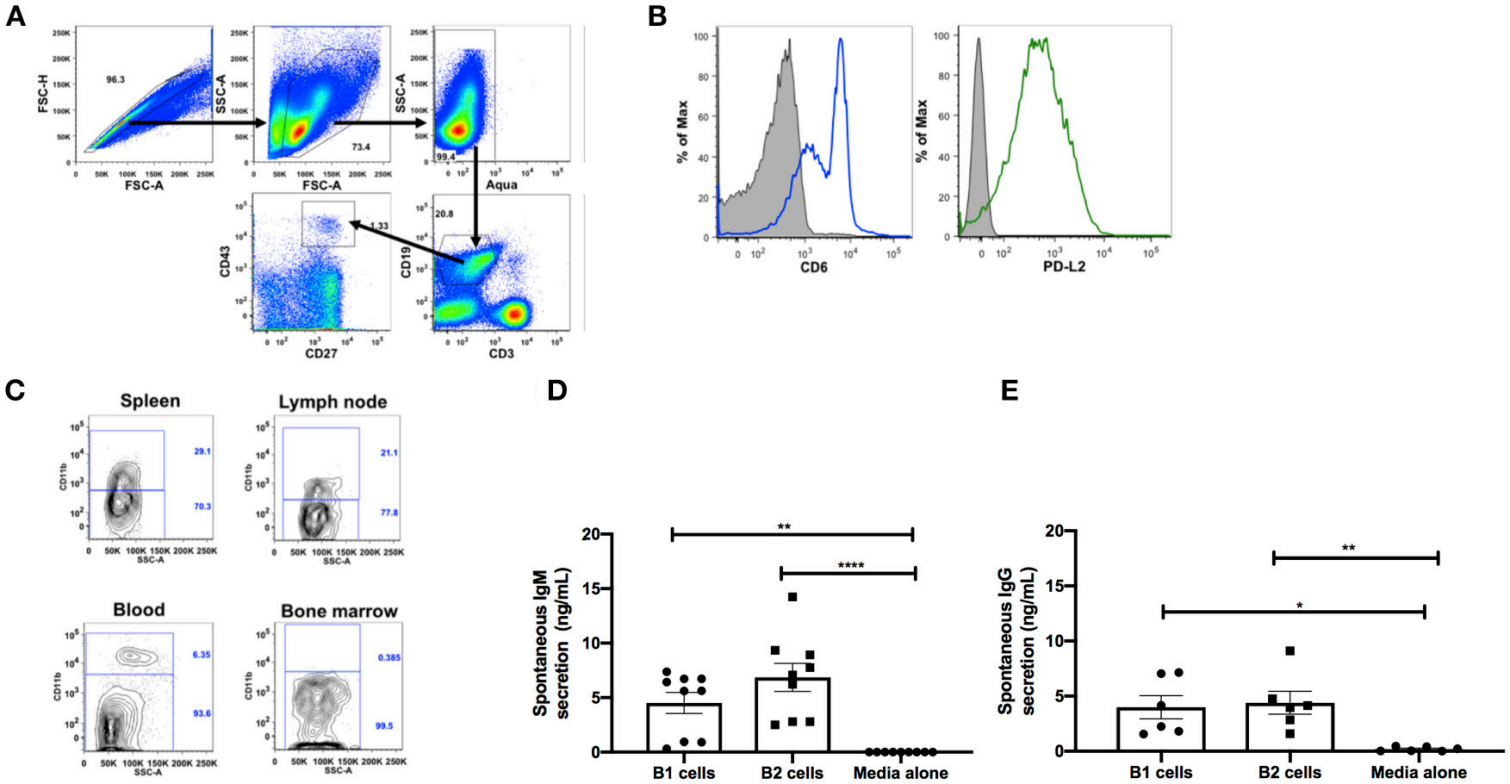
Phenotypic and functional characterization of B1 cells in rhesus macaques. All samples were obtained from naïve macaques except for the spleen samples in **(C)** which were from chronically SIV-infected macaques. **(A)** Flow cytometry gating strategy for human-like B1 cells, defined as CD3^−^CD19^+^CD43^+^CD27^+^. **(B)** Histogram analysis of CD6 and PD-L2 expression on B1 cells in PBMC. Isotype control is shown in solid gray. Graph is representative of 10 macaques. **(C)** The contour plot displays expression of CD11b by gating on CD3^−^CD19^+^CD27^+^CD43^+^ cells. Data are representative of 15 macaques. Flow sorted CD19^+^CD27^−^CD43^−^ (B2 cells) and CD19^+^CD27^+^CD43^+^ (B1 cells) from naive PBMC were cultured for 3 d, after which supernatants were evaluated for secretion of **(D)** IgM and **(E)** IgG by ELISA. Data are from nine macaques for IgM and six macaques for IgG. For statistical analysis, nonparametric Kruskal–Wallis and Dunn's pairwise multiple comparison tests were performed. All tests were two tailed. *p < 0.05, **p < 0.01, ****p < 0.0001.

To explore the potential role of B1 cells in the pathogenesis of SIV infection, we first investigated whether B1 cells exist in rhesus macaques, the most commonly used non-human primate model for HIV research. Using a panel of phenotypic markers recently reported to be expressed by human B1 cells (6), we identified a similar CD3^−^CD19^+^CD43^+^CD27^+^ cell population in the peripheral blood of rhesus macaques (Figure 1A). Additionally, these B1 cells were also present in the spleen, LN, and bone marrow as expected (Table 1). Surprisingly, given that B1 cells are predominantly abundant in the peritoneal cavity of mice as well as present in the skin of humans and mice (1, 29), B1 cells were not observed in the peritoneal cavity and rectal pinches of the rhesus macaques (Table 1).

**Table 1:** Tissue distribution of B1 cells in rhesus macaques^a^.

**Tissue**	**B1 cell frequency^b^ (Mean ± SEM)**	**B1 cell frequency range (Minimum–Maximum)**	**Number of macaques investigated**
PBMC	5.34 ± 0.72	3.28–10.50	10
Spleen	2.58 ± 0.47	0.82–11.70	25
Lymph node	0.56 ± 0.10	0.28–1.00	7
Bone marrow	9.80 ± 5.30	1.86–30.80	5
Peritoneal lavage	Undetected	Undetected	4
Rectal Pinches	Undetected	Undetected	4

a *Naive macaques were used in assessing B1 cell tissue distribution except for the spleen. Spleens from chronically infected macaques were used as naïve spleens were not available*.

b *B1 cell frequency is the percentage of CD43^+^CD27^+^ within the CD19^+^ cell population*.

To further determine if the B1 cell population in rhesus macaques is phenotypically similar to that found in mice, we examined the surface expression levels of CD6 and PD-L2 which are specifically expressed on mouse B1 cells (13, 30). Both surface markers were also expressed on B1 cells from rhesus macaque PBMC (Figure 1B). Human and mouse B1 cells can be further divided into two distinct subsets based on the CD11b surface antigen (4, 6, 12, 31). We found both CD11b^+^ and CD11b^−^ B1 cell subsets in macaque PBMC, spleen, and LN; CD11b^+^ cells were not present in bone marrow (Figure 1C). The inherent ability to spontaneously secrete IgM is the hallmark functional characteristic of B1 cells in both humans and mice (1, 6). Therefore, we sorted B1 cells and CD3^−^CD19^+^CD43^−^CD27^−^ (B2 cells) from the peripheral blood of naïve rhesus macaques and assessed their spontaneous IgM secretion *in vitro* over a 3 day period by ELISA. The sorted B1cells secreted significantly more IgM *in vitro* compared to controls (media alone) (Figure 1D). Surprisingly, the B2 cells also spontaneously secreted IgM compared to controls. Although the B2 cells secreted more IgM compared to the B1 cells, this difference was not significant (Figure 1D). To determine whether the spontaneous secretion of antibodies by the sorted B1 cell population was primarily restricted to the IgM subclass, we also measured the ability of these cells to secrete IgG. We found that B1 and B2 cells also spontaneously secreted IgG *in vitro* (Figure 1E). There was no significant difference between B1 and B2 cells with regard to spontaneous IgG secretion. In sum, B1 cells displaying phenotypic and functional characteristics similar to mouse and human B1 cells are present in rhesus macaques.

### Effect of SIV Infection on the Dynamics of B1 Cells in the Blood, LN, and Bone Marrow

**Figure 2:**
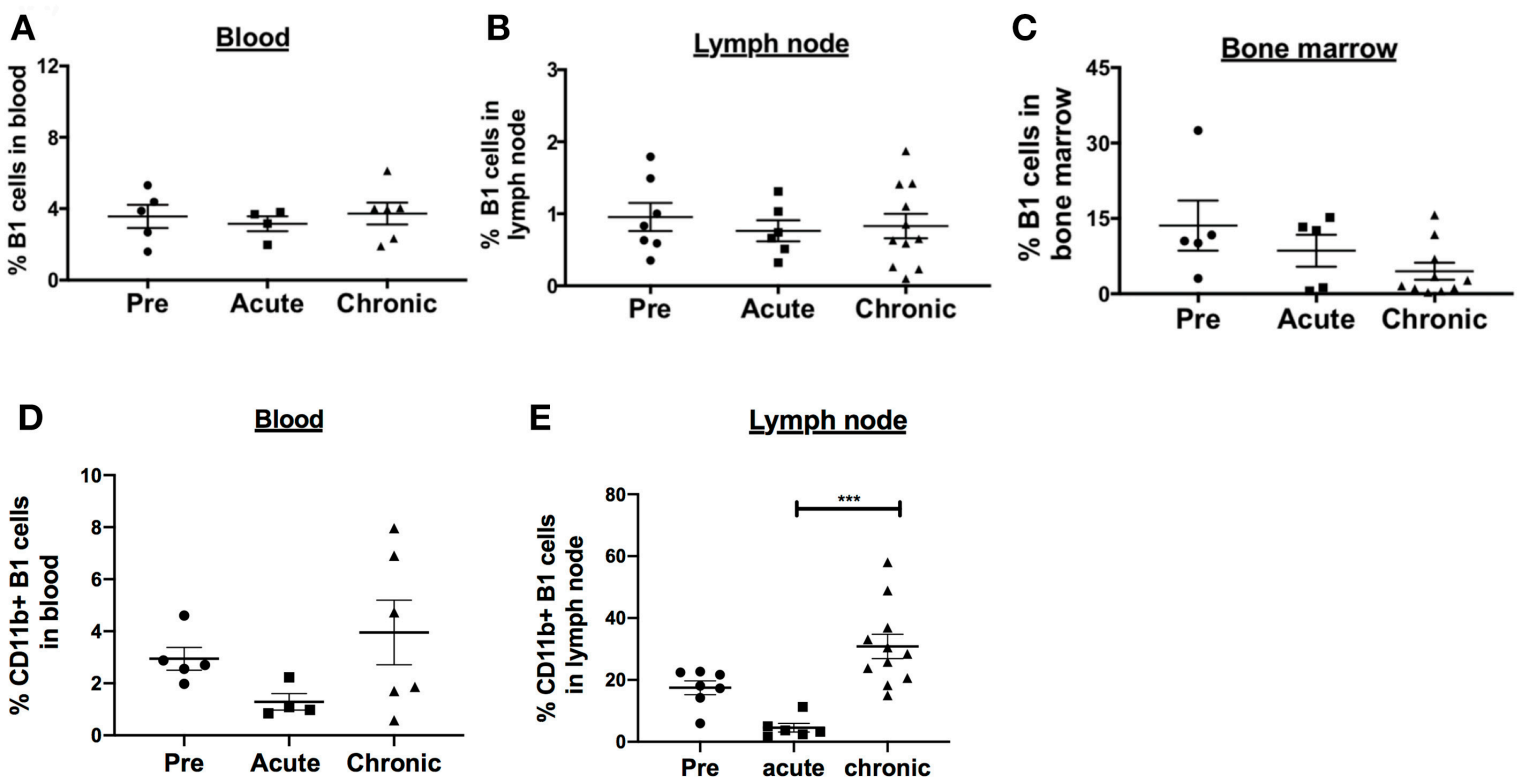
Effect of SIV infection on B1 cell populations in rhesus macaques. The frequency of B1 cells was analyzed by flow cytometry in the **(A)** blood, **(B)** LN, and **(C)** bone marrow of three groups of macaques: uninfected (pre), and acutely and chronically SIV infected. Data for blood are from 4 to 6 macaques, LN from 6 to 11 macaques, and bone marrow from 5 to 10 macaques. The frequency of CD11b^+^ B1 cells was also analyzed in the **(D)** blood and **(E)** LN of the three macaques groups. The frequency of CD11b^+^ B1 cells is the percentage of CD11b^+^ cells within the CD19^+^CD43^+^CD27^+^ cell population. Data are shown as means ± SEM. For statistical analysis, non-parametric Kruskal–Wallis and Dunn's pairwise multiple comparison tests were performed. All tests were two tailed. ***p < 0.001.

SIV infection is associated with activated memory B cell depletion and dysfunction in the peripheral blood and LN of rhesus macaques (15, 32, 33). To determine the effect of SIV infection on the frequency of B1 cells and their activation state, we performed a cross-sectional analysis of rhesus macaques categorized as uninfected, acutely-infected or chronically-infected. The B1 cell populations in the peripheral blood and LN were not significantly altered during acute and chronic infection compared to pre-infection levels (Figures 2A,B). In the bone marrow, the frequency of B1 cells decreased in the acute and chronic groups, however not significantly compared to the pre-infection group (Figure 2C). Next, we examined the effect of SIV infection on the CD11b^+^ B1 cell subset which is recruited to the LNs of mice following viral infection (4). The frequency of CD11b^+^ B1 cells in the blood was decreased in the acute group and recovered to the pre-infection level in the chronically-infected group, however these changes were not significant (Figure 2D). In contrast, in the LN the CD11b^+^ B1 cell subset decreased in the acutely-infected animals, but significantly expanded in the chronically-infected macaques compared to the acute group (Figure 2E). The dynamics of B1 cells and CD11b^+^ B1 cell subsets in the spleen were not analyzed due to lack of pre-infection spleen samples. In sum, the CD11b^+^ B1 cells in the LN were elevated in the group of chronically-infected macaques suggesting a potential role for these cells in SIV pathogenesis.

### Effect of SIV Infection on B1 Cell Activation

**Figure 3:**
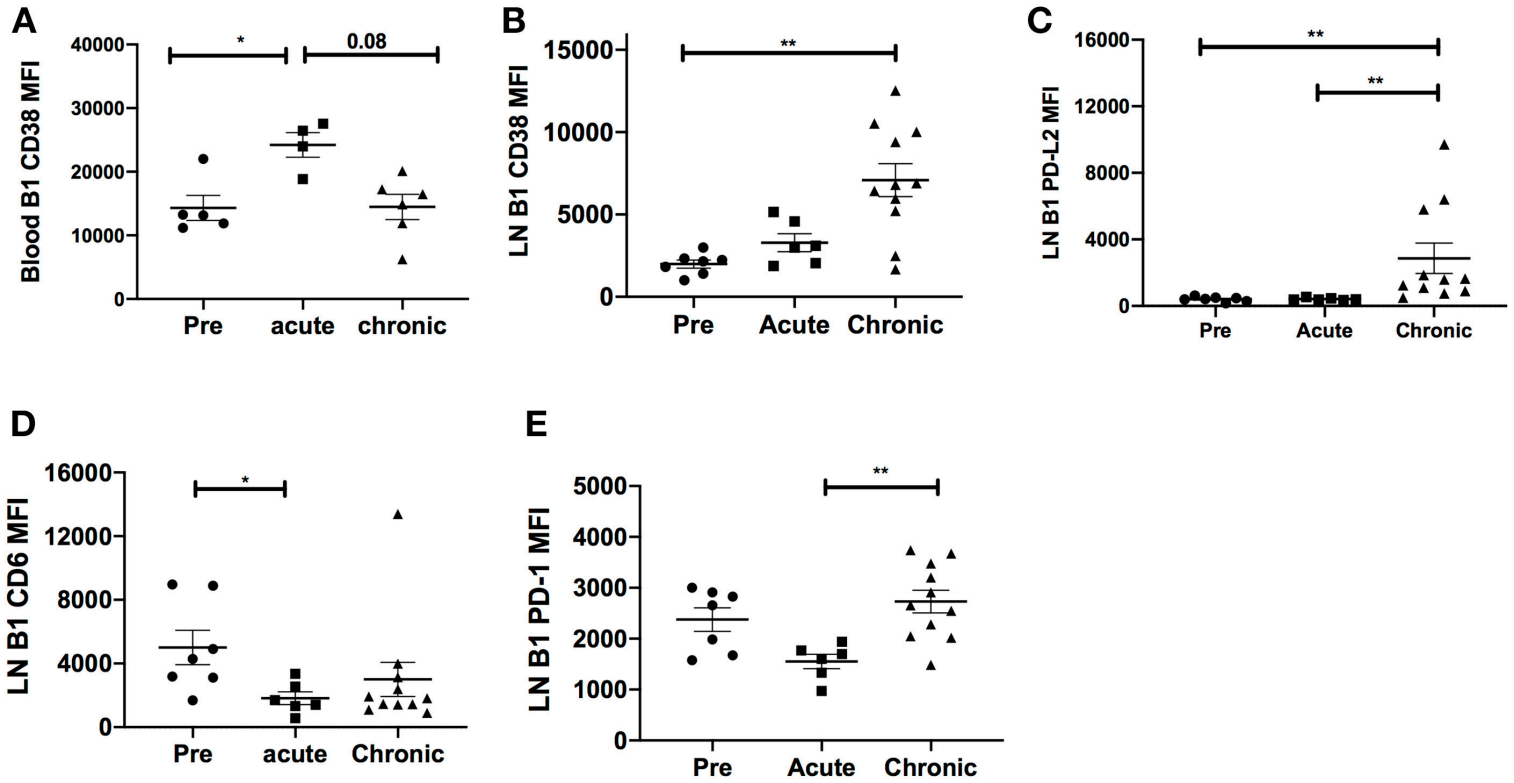
Effect of SIV infection on B1 cell activation state. The expression levels of CD38 were analyzed on B1 cells in the **(A)** blood and **(B)** LN of three macaque groups: uninfected (pre), and acutely and chronically SIV infected. The expression levels of **(C)** PD-L2, **(D)** CD6, and **(E)** PD-1 were analyzed on B1 cells in the LN in the three macaque groups. Displayed are the mean fluorescence intensities (MFI) of the surface markers analyzed. Data for the blood CD38 MFI are from 4 to 6 macaques, and for LN CD38, PD-L2, CD6, and PD-1 are from 6 to 11 macaques. Data are reported as means ± SEM. For statistical analysis, non-parametric Kruskal–Wallis and Dunn's pairwise multiple comparison tests were performed. All tests were two-tailed. *p < 0.05, **p < 0.01.

It was possible that the expansion of LN CD11b^+^ B1 cells might be driven by overall chronic activation of the B1 cell population. Therefore, we analyzed the surface expression of several costimulatory and inhibitory molecules on B1 cells. In blood, the B cell activation molecule, CD38, was significantly upregulated in the acutely-infected animals but returned to basal levels in the chronically-infected group indicating a transient activation of B1 cells (Figure 3A). In the LN, the expression levels of CD38 and PD-L2 were significantly upregulated in chronically-infected macaques compared to pre-infection levels (Figures 3B,C). The expression levels of CD6 were significantly reduced in acutely-infected macaques compared to pre-infection levels and the PD-1 expression level was significantly elevated in the chronically-infected group compared to the acutely infected group. However, the expression levels of these two molecules between the chronic and pre-infection groups were not significantly different (Figures 3D,E). We also assessed the frequency of these activation markers in the blood and LN. The frequency of CD38-expressing B1 cells in the blood was not significantly altered following SIV infection (Supplementary Figure 1A). In the LN, we found that the frequencies of CD38 and PD-L2-expressing B1 cells were significantly elevated in chronically-infected macaques compared to naïve or acutely-infected macaques (Supplementary Figures 1B,C). Additionally, we found that the frequency of CD6-expressing B1 cells was significantly reduced in the LN of acutely-infected macaques compared to uninfected macaques (Supplementary Figure 1D). Finally, the frequency of PD-1 expressing B1 cells in the LN was significantly elevated in chronically-infected macaques compared to acutely-infected macaques; however, it was not significantly altered compared to uninfected animals (Supplementary Figure 1E). In sum, B1 cells in the blood and LN displayed enhanced immune activation following SIV infection. However, the activation was not sufficient to induce B1 cell exhaustion and population depletion, consistent with the lack of PD-1 upregulation which has been associated with loss of memory B cells in rhesus macaques (15).

### B1 Cell Frequency Correlates Positively With Markers of SIV Disease Progression

**Figure 4:**
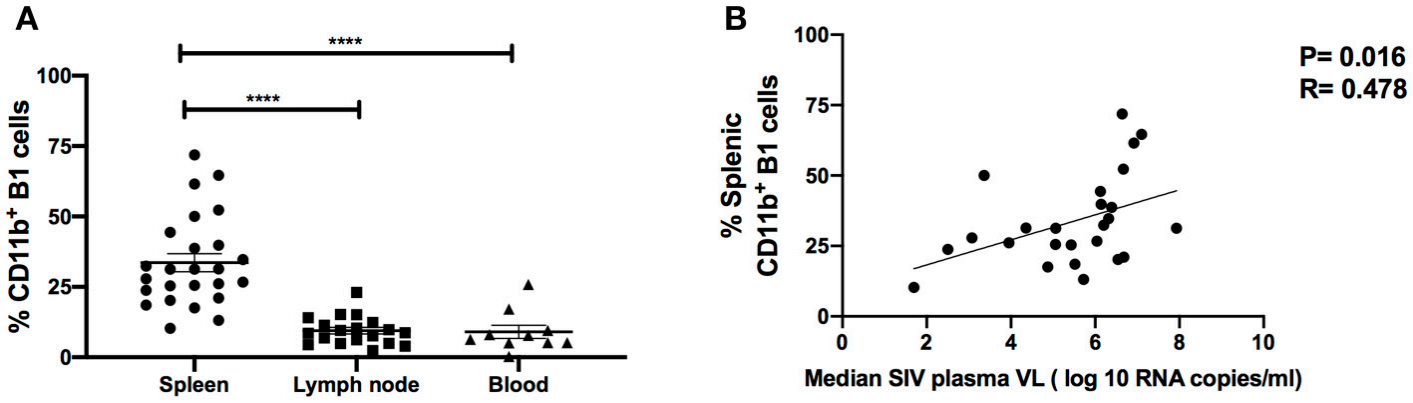
CD11b^+^ B1 cells are abundant in the spleen and correlate with SIV viral load in the chronic phase of infection. **(A)** The frequency of CD11b^+^ B1 cells was analyzed in the spleen, LN, and blood during the chronic phase of SIV infection. Data (means ± SEM) are shown for spleen (25 macaques), LN (11 macaques), and blood (10 macaques). For statistical analysis, non-parametric Kruskal–Wallis and Dunn's pairwise multiple comparison tests were performed. **(B)** The correlation of CD11b^+^ B1 cells with the median SIV plasma viral load (VL) over weeks 12–40 post-infection. Data are from 25 macaques. For statistical analysis, the non-parametric Spearman rank correlation was performed. All tests were two-tailed. ****p < 0.0001.

To investigate the potential role of B1 cells in SIV disease progression, we assessed relationships between the frequency of B1 cells, SIV plasma viral load and the frequency of exhausted T cells. The frequency of B1 cells in the blood, LN and bone marrow during the chronic phase of infection did not correlate with SIV plasma viral load (data not shown), however we found that the frequency of splenic B1 cells tended to correlate positively with SIV viral load (*r* = 0.33, *p* = 0.11). This finding prompted us to further characterize splenic B1 cells. We hypothesized that the tendency of splenic B1 cells to correlate with SIV plasma viral load might be driven by their subset composition. We compared the frequency of CD11b^+^ B1 cells within the spleen, LN and blood given that this B1 cell subset is associated with viral infection (4) and was expanded in the LN during SIV infection (Figure 2E). The frequency of CD11b^+^ B1 cells was highest in the spleen of chronically SIV-infected macaques compared to the LN and blood (Figure 4A). Additionally, we found a significant positive correlation between the frequency of splenic CD11b^+^ B1 cells and SIV plasma viral load (Figure 4B).

**Figure 5:**
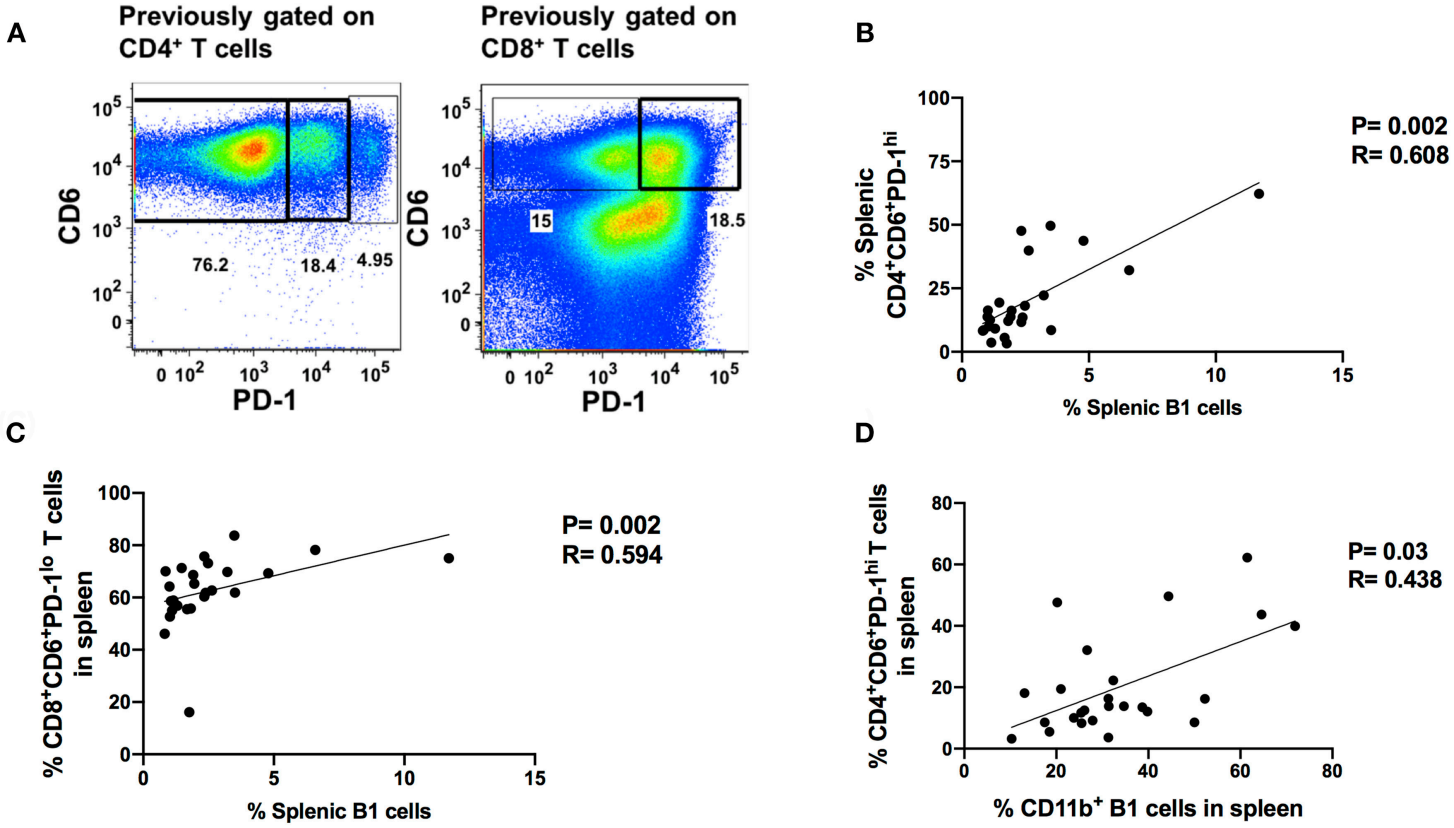
Splenic B1 cells correlate with T cell exhaustion during chronic SIV-infection. **(A)** Flow cytometric analysis of chronically SIV-infected splenic exhausted T cells by gating on CD4^+^CD6^+^PD-1^+^ and CD8^+^CD6^+^PD-1^+^ respectively. Data are representative of 25 macaques. For CD4^+^ T cells: CD6^+^PD-1^−^ (non-exhausted), CD6^+^PD-1^lo^ (exhausted), and CD6^+^PD-1^hi^ (terminally exhausted). For CD8^+^ T cells: CD6^+^PD-1^−^ (non-exhausted) and CD6^+^PD-1^+^ (exhausted). **(B)** The correlation of splenic B1 cells with terminally exhausted CD4^+^ T cells. **(C)** The correlation of splenic B1 cells with exhausted splenic CD8^+^ T cells. **(D)** The correlation of splenic CD11b^+^ B1 cells with terminally exhausted CD4^+^ T cells. Data in **(B–D)** are from 25 macaques. For statistical analysis, non-parametric Spearman rank correlations were performed. All tests were two-tailed.

The progressive loss of T-cell antiviral activity in HIV and SIV infection due to exhaustion is associated with increased viral burden (17, 23). Therefore, we explored the relationship between B1 cells and exhausted T cells as a potential mechanism by which B1 cells may enhance SIV plasma viral load given that: (1) B1 cells are potent modulators of T cell immune responses (12, 14, 34), and (2) B1 cells express PD-1 ligands. We utilized the co-expression of PD-1 and CD6 to define exhausted/dysfunctional T cells in chronically infected macaques (Figure 5A). We identified two subpopulations of exhausted CD4^+^ T cells that differentially express PD-1 during chronic infection. PD-1^lo^ cells have been shown to be responsive to anti-PD-L1 blockade, whereas PD-1^hi^ cells respond poorly to PD-1 blockade and appear to be terminally differentiated (35). We observed only one population of exhausted splenic CD8^+^ T cells, PD-1^lo^. Additionally, LAG-3 has been previously reported to be expressed on exhausted T cells (36, 37). Therefore, we also assessed its expression on these CD6 and PD-1 co-expressing CD4^+^ and CD8^+^ T cells in chronically-infected macaques. We found that LAG-3 was also expressed on CD4^+^ and CD8^+^ T cells co-expressing CD6 and PD-1 (Supplementary Figure 2A). Exhausted T cells are associated with elevated HIV/SIV plasma viral load; therefore, we assessed the correlation between the frequency of splenic CD4^+^CD6^+^PD-1^lo^, CD4^+^CD6^+^PD-1^hi^, and CD8^+^CD6^+^PD-1^lo^ T cells with the median chronic SIV plasma viral load. The frequency of CD4^+^CD6^+^PD-1^hi^ and CD8^+^CD6^+^PD-1^lo^ T cells positively correlated with the median SIV plasma viral load (Supplementary Figures 2B,C) suggesting a potential dysfunctional state, whereas the frequency of splenic CD4^+^CD6^+^PD-1^lo^ did not (Supplementary Figure 2D) as expected.

Here, we examined the association of B1 cells with exhausted PD-1^hi^ CD4^+^ T cells and PD-1^lo^ CD8^+^ T cells. The frequency of B1 cells in the blood, bone marrow and LN did not correlate with the frequency of exhausted T cells, either CD4^+^ or CD8^+^ (data not shown). However, the frequency of B1 cells in the spleen positively correlated with the frequency of exhausted PD-1^hi^CD4^+^ and PD-1^lo^ CD8^+^ T cells (Figures 5B,C). The frequency of splenic CD11b^+^ B1 cells also correlated with the frequency of terminally exhausted PD-1^hi^ CD4^+^ T cells (Figure 5D). These data suggest that B1 cells may contribute to T cell exhaustion in the spleen and that the CD11b^+^ subpopulation may specifically contribute to the terminal exhaustion of CD4^+^ T cells. Given that CD4^+^ T cells play an important role in viral control by eliciting and/or maintaining optimal anti-viral CD8^+^ T cell responses and these responses are impaired during exhaustion, our data further suggest that CD11b^+^ B1 cells may enhance SIV plasma viral load by indirectly contributing to CD4^+^ T cell exhaustion.

### B1 Cells Possess T Cell Exhaustion-Sustaining Capabilities

**Figure 6:**
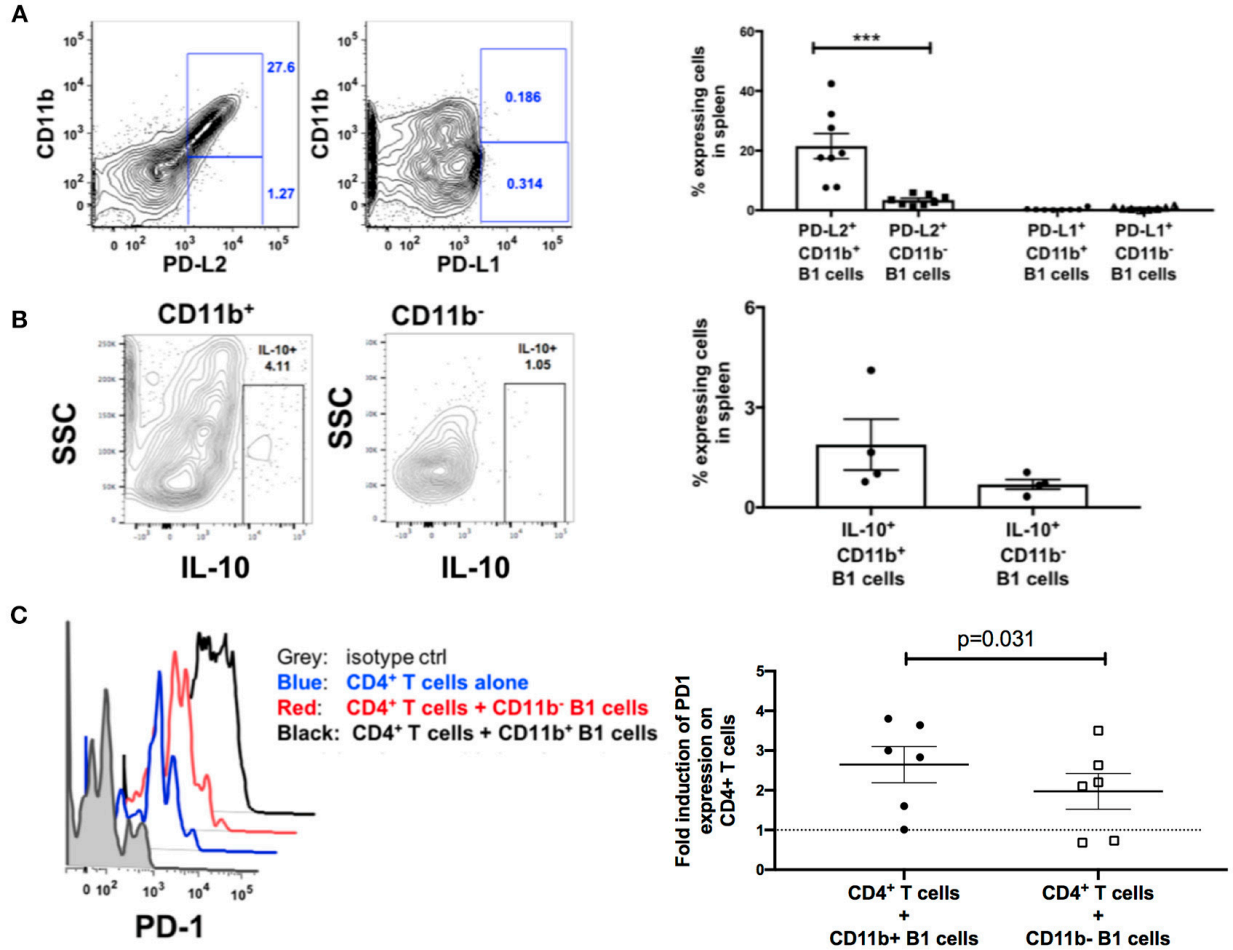
B1 cells possess T-cell exhaustion-inducing potential. **(A)** Representative example of flow cytometric analysis of PD-L1 and PD-L2 expression on splenic B1 cells by gating on CD3^−^CD19^+^CD43^+^CD27^+^ CD11b^+^ or CD3^−^CD19^+^CD43^+^CD27^+^ CD11b^−^ cells. Shown to the right are mean frequencies ± SEM. Data are from 8 macaques. **(B)** Representative example of flow cytometric analysis of IL-10^+^ splenic B1 cells by gating on CD3^−^CD19^+^CD43^+^CD27^+^ CD11b^+^ or CD3^−^CD19^+^CD43^+^CD27^+^ CD11b^−^ cells. Shown to the right are mean frequencies ± SEM. Data are from 4 macaques. Splenocytes were acquired during the chronic phase of SIV infection. For statistical analysis nonparametric unpaired Mann–Whitney tests were performed. ***p < 0.001. **(C)** Flow cytometric analysis of PD-1 expression on splenic CD3^+^ CD4^+^ T cells following *in vitro* co-culture with sorted CD11b^+^ or CD11b^−^ B1 cells. Gray: PD-1 isotype control, Blue (PD-1 expression on CD4^+^ T cells alone), Red (PD-1 expression on CD11b^−^ B1 cells + CD3^+^CD4^+^ T cells), and Black (PD-1 expression on CD11b^+^ B1 cells + CD3^+^CD4^+^ T cells). Shown to the right is the fold-induction of PD-1 expression on CD3^+^CD4^+^ T cells. Data are from six macaques. For statistical analysis, Wilcoxon signed rank tests were performed. The dashed horizontal line at 1 indicates the null value.

Engagement of PD-1 by its ligands PD-L1 or PD-L2 transduces a signal that inhibits T-cell activation, proliferation, cytokine secretion, and cytolytic function. Therefore, we hypothesized that splenic B1 cells may contribute to T cell exhaustion via PD-1 and PD-L1/PD-L2 engagement. We characterized the expression of PD-1 ligands on splenic B1 CD11b^+^ and CD11b^−^ subsets. Splenic B1 cells specifically expressed PD-L2, and its expression was predominantly restricted to the CD11b^+^ B1 cell subset (Figure 6A) indicating a potential mechanism by which splenic B1 cells may induce T cell exhaustion. B1 cells in humans and mice also spontaneously produce IL-10 which has been reported to induce T cell exhaustion (12, 29, 38–40). Therefore, we investigated whether splenic B1 cells in rhesus macaques also express IL-10 as a potential alternate mechanism of contributing to T cell exhaustion. We found a population of IL-10^+^ splenic B1 cells during the chronic phase of SIV infection by intracellular cytokine staining (Figure 6B). The mean frequency of IL-10^+^ splenic B1 cells was 2.7-fold higher within the CD11b^+^ subset compared to the CD11b^−^ B1 cell subset (Figure 6B), which is consistent with previous studies in humans (12). Elevated expression of PD-1 is associated with T cell exhaustion, therefore we investigated whether sorted splenic CD11b^+^ B1 cells from the chronic phase of SIV infection could directly influence PD-1 expression on T cells in an *in vitro* co-culture assay. We discovered that splenic CD11b^+^ B1 cells were able to significantly influence PD-1 upregulation on splenic CD4^+^ T cells compared to their CD11b^−^ B1 cell counterparts (Figure 6C). Collectively, these data suggest that splenic CD11b^+^ B1 cells possess the ability to influence T cell exhaustion and could directly contribute to elevated PD-1 expression on T cells during the chronic phase of SIV infection.

## Discussion

We investigated the presence of B1 cells in rhesus macaques and performed a cross-sectional study to address their role in SIV infection given that (1) B1 cells are potent modulators of T cell effector functions and (2) natural antibodies secreted by B1 cells share polyreactive or autoreactive features with several HIV-1 broadly neutralizing antibodies. We hypothesized that B1 cells would play a protective role in SIV disease progression. In this study, we report the presence of human-like B1 cells in rhesus macaques as shown by surface marker expression and spontaneous IgM secretion *in vitro*. In response to SIV infection, the B1 cell population in rhesus macaques remained unperturbed, except for the CD11b^+^ B1 cell subset that was significantly expanded in the peripheral lymphoid tissue of the chronically-infected macaques. The frequency of the splenic CD11b^+^ B1 cell subset positively correlated with the SIV plasma viral load and the frequency of exhausted T cells. Thus, rather than a protective role, we found that a subset of B1 cells appeared to negatively impact SIV disease progression. Mechanistically, we found that splenic B1 cells expressed IL-10 and PD-L2 and were able to directly influence PD-1 upregulation on CD4^+^ T cells *in vitro*.

A previous study investigated the presence of B1 cells in non-human primates using characteristics unique to murine B1 cells (5). The results indicated that mouse B1-like IgM^+^CD19^+^CD11b^+^ cells are present in African green monkeys (AGM) and cynomolgus macaques (CM). How these AGM and CM B1-like cells relate to the human CD19^+^CD43^+^CD27^+^ B1 cells recently described by Rothstein et al. (6) in terms of functionality and potential role in the pathogenesis of SIV infection was not addressed. In this study, we identified CD19^+^CD43^+^CD27^+^ cells in rhesus macaques. These B1 cells expressed the surface markers CD6, PD-L2, and CD11b, the expression of which is restricted to B1 cells in mice and humans (6, 12, 13, 30, 31). Functionally, these human-like B1 cells possessed the ability to spontaneously secrete IgM *in vitro*; a hallmark function of B1 cells in mice and humans. Additionally, we observed that B1 cells in rhesus macaques also possessed the ability to spontaneously secrete IgG *in vitro*. This finding is consistent with a recent study indicating that proposed human B1 cells could spontaneously secrete IgA, IgG, and IgM due to their pre-plasmablast phenotype (41). Additionally, the spontaneous secretion of IgG could be due to the presence of class switched B1 cells. Previous studies in mice have reported that although B1 cells express IgM, they can class switch to IgG or other isotypes (42–45). Surprisingly, B2 cells also secreted IgM in contrast to previous studies in mice and humans (6). The spontaneous IgM secretion by the non-B1 cells may be due to the presence of CD27^−^ plasma cells/plasma blasts identified in rhesus macaques (32). In this previous report, only the ability of the CD27^−^ B cells to secrete IgA and IgG was investigated. Our data confirm the ability of CD27^−^ B cells to spontaneously secrete IgG and suggest that these CD27^−^ B cells may also possess the ability to spontaneously secrete IgM. Collectively, our data indicate that rhesus macaques have a population of B cells with defining characteristics of human and mouse B1 cells.

The tissue distribution of B1 cells in rhesus macaques was distinctly different from AGM, CM, and mice. In contrast to these species where B1 cells are predominantly enriched in the peritoneal lavage and omental tissues (5, 31, 34), we were unable to detect human-like B1 cells in these tissues of rhesus macaques, suggesting that rhesus B1 cells may be primarily located in a reservoir other than the peritoneal cavity. The bone marrow may serve as such a reservoir given that: (1) B1 cells were predominantly located in the bone marrow compared to other tissues surveyed, and (2) B1 and B2 cells in humans arise from a common bone marrow progenitor subset (46). Future work is necessary to assess the presence and tissue distribution of this hematopoietic stem cell subset in rhesus macaques.

SIV infection causes depletion of naïve and memory B cells in rhesus macaques (15, 32, 33, 47, 48). This loss of memory B cells is associated with elevated B cell immune activation. The total B1 cell population in the PBMC, LN and bone marrow was not significantly reduced during the course of SIV infection indicating that B1 cells may not be as susceptible to depletion as memory B cells. Although, several B cell activation markers such as CD38 and PD-L2 were significantly elevated in terms of expression levels and frequency on B1 cells following SIV infection, the threshold of activation may not have been sufficient to induce B1 cell exhaustion and depletion. PD-1 overexpression has been associated with loss of memory B cells in rhesus macaques (15). Here, although PD-1 expression was elevated in the chronic infection group compared to the acute infection group (Figure 3E), it did not differ from pre-infection levels, consistent with the observed lack of B1 cell loss or depletion. While the total B1 cell population was unchanged, the CD11b^+^ B1 cell subset was expanded in the LN of chronically-infected macaques compared to that of acutely-infected macaques (Figure 2E). The elevated frequency of CD11b^+^ B1 cells in chronically-infected macaques compared to uninfected or acutely-infected macaques suggests a role for these cells in SIV pathogenesis.

Several studies in humans and rhesus macaques have suggested that the presence of a functional spleen contributes to HIV/SIV disease progression (49–53). Splenectomized rhesus macaques have lower viral burdens and longer survival than unsplenectomized controls following SIV infection (51). In a similar study in humans, a rapid and sustained increase of CD4^+^ T cells in HIV-1-seropositive patients was observed following splenectomy (50, 53, 54). These studies suggest the presence of host factors within the spleen that may enhance HIV/SIV disease progression. We observed a positive correlation between B1 cells and SIV plasma viral load suggesting a pathogenic role for B1 cells during SIV infection. This positive correlation was restricted to splenic B1 cells and appeared to be driven specifically by the CD11b^+^ B1 cell subset (Figure 4B). Several studies have shown that B1 cells can either enhance or suppress T cell immune activity (3, 6, 12, 13, 34, 55). This suggests that B1 cells can heavily influence the nature and direction of T cell antiviral responses resulting either in elevated or decreased viral loads during the course of SIV infection. Therefore, we assessed the correlation between B1 cells and exhausted T cells. The splenic B1 cells positively correlated with exhausted T cells (Figures 5B,C) and this association was also driven by the CD11b^+^ B1 cell subset (Figure 5D). These data suggest that B1 cells may contribute to T cell exhaustion resulting in elevated SIV viral burdens.

To explore the potential mechanism(s) by which B1 cells may contribute to T cell exhaustion *in vivo*, we first characterized the expression of PD-1 ligands (PD-L1 and PD-L2) on splenic B1 cells given that elevated PD-1 expression is the hallmark of T cell exhaustion. Both PD-L1 and PD-L2 have been reported to be expressed on mouse B1 cells (13, 14); however, their expression on human B1 cells as well as possible subset specific expression is unclear. We found that PD-L2 was specifically expressed on rhesus macaque B1 cells and its expression was restricted to the CD11b^+^ B1 cell subset (Figure 6A) indicating a potential mechanism of suppressing anti-viral T cell effector functions *in vivo* and enhancing SIV plasma viral load. In addition to the potential receptor/ligand interaction, we found that splenic CD11b^+^ B1 cells could also express IL-10 (Figure 6B) which has been implicated in impaired anti-viral T cell effector function and elevated viral load. These novel findings suggest that splenic B1 cells could enhance SIV viremia by influencing anti-viral T cell effector function potentially via either the PD-L2/PD-1 or IL-10 pathway. Additionally, we discovered that splenic CD11b^+^ B1 cells from SIV chronically-infected macaques could directly influence PD-1 upregulation on CD4^+^ T cells *in vitro* (Figure 6C). These findings are consistent with previous studies indicating that CD11b^+^ B1 cells are potent stimulators of T cell activation and expansion compared to their CD11b^−^ B1 cell counterparts (6). The potency of CD11b^+^ B1 cells in stimulating T cells was attributed in part to the constitutively elevated expression of CD80 and CD86. It is possible that the elevated expression of these co-simulatory molecules on CD11b^+^ B1 cells may also play a role in the chronic stimulation of T cells resulting in T cell exhaustion during the chronic phase of SIV infection.

In summary, based on the data provided in this study, we show that B1 cells with phenotypic and functional characteristics similar to those of human B1 cells are present in rhesus macaques. We discovered a potential pathogenic role for splenic B1 cells in SIV disease progression. The association of B1 cells with SIV viremia was attributed to the CD11b^+^ B1 cell subset. The induction of T cell activation and differentiation by these cells may be the mechanism by which B1 cells influence SIV viremia control. In view of the observations discussed above concerning splenectomized HIV-infected patients and SIV-infected macaques, it would be interesting to investigate the effect of the absence of splenic B1 cells on viral burden and CD4^+^ T cell counts in splenectomized HIV-infected individuals. Understanding the role of these unique innate B cells may lead to a novel therapeutic target for impeding or even preventing HIV/SIV disease progression.

## Data Availability

The datasets generated for this study are available on request to the corresponding author.

## Author Contributions

GE-A and MR-G developed the concept and designed the experimental plans. GE-A, AN, and CH performed the experiments. GE-A, AN, MR, SH, E-JK, RH, and TH processed tissue samples and provided data critic. GE-A and AN analyzed the data. DJV conducted statistical analysis. GE-A and MR-G prepared the manuscript. All authors reviewed the manuscript.

### Conflict of Interest Statement

The authors declare that the research was conducted in the absence of any commercial or financial relationships that could be construed as a potential conflict of interest.
