# Supplementary material for: A Pathogenic Role for Splenic B1 Cells in SIV Disease Progression in Rhesus Macaques
Source: Front Immunol. 2019 Mar 19;10:511. doi: 10.3389/fimmu.2019.00511 (PMC6433970; doi:10.3389/fimmu.2019.00511)
Supplement: Supplementary file 1 [file Data_Sheet_1.PDF]

Supplementary Figure 1

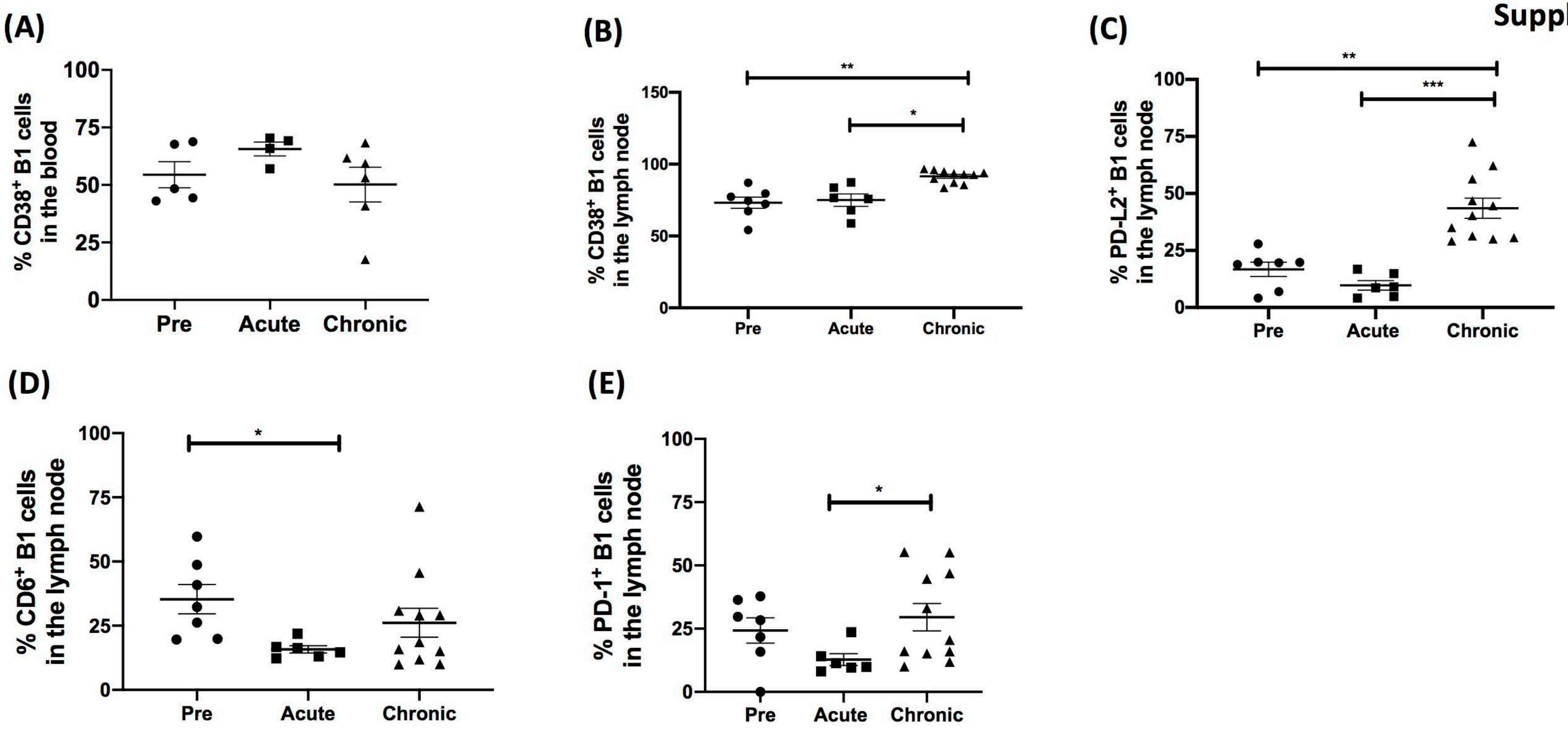

Supplementary Figure 1. Effect of SIV infection on the B1 cell activation state (frequency). The frequency of CD38 expressing-B1 cells in the (A) blood and (B) LN in three macaque groups: uninfected (pre), and acutely and chronically SIV infected. The frequency of (C) PD-L2, (D) CD6 and (E) PD-1 expressing B1 cells were analyzed in the LN of the same three groups. Data for the blood CD38 frequency are from 4-6 macaques, LN CD38, PD-L2, CD6 and PD-1 are from 6-11 macaques. Data are reported as means ± SEM. For statistical analysis, non-parametric Kruskal-Wallis tests were performed. All tests were two-tailed. *p<0.05, ** p<0.01,***p<0.001.

Supplementary Figure 2

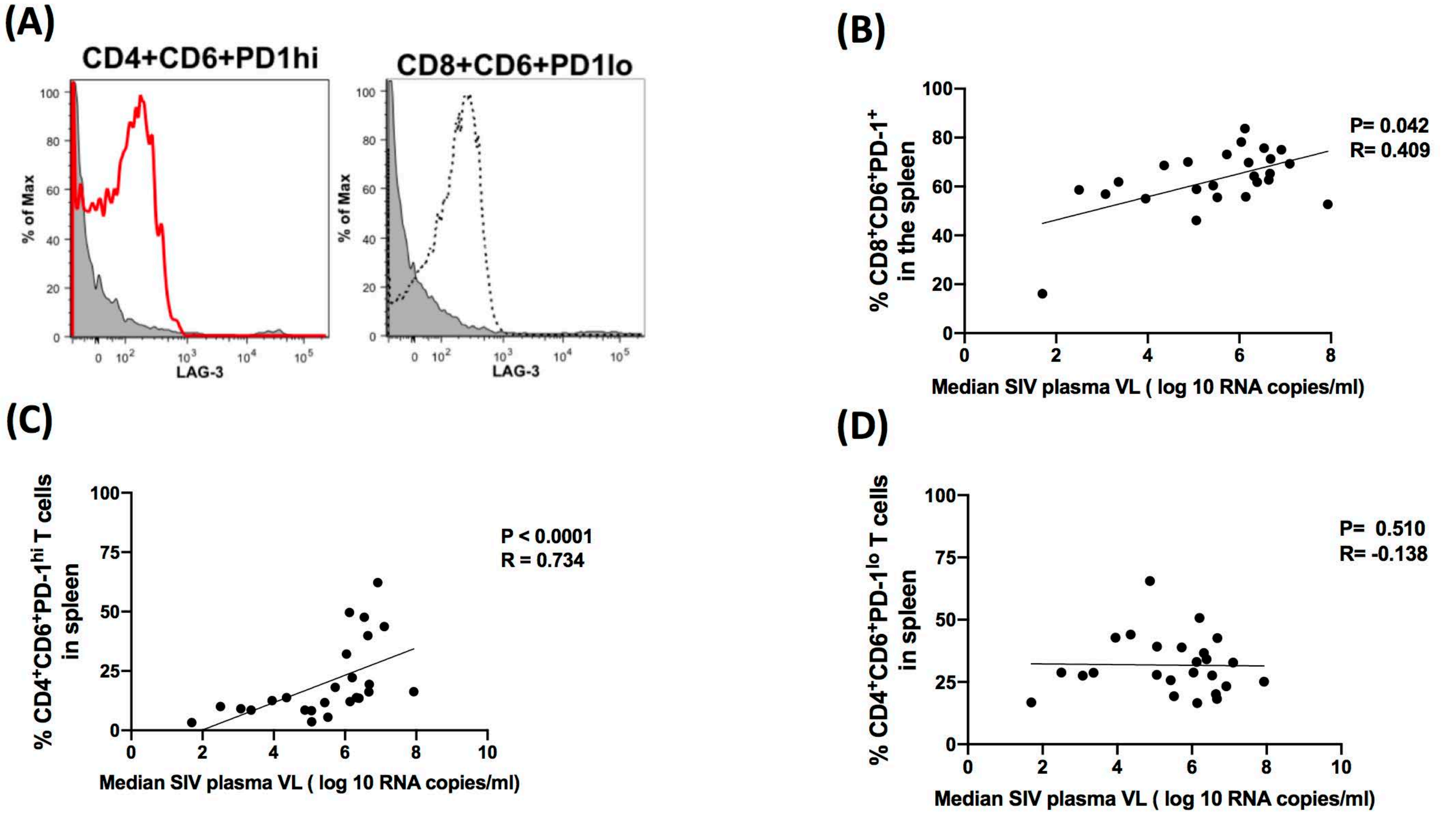

Supplementary Figure 2. Characterization of exhausted T cells during chronic SIV infection. (A) Representative example of flow cytometric analysis of LAG-3 expression on splenic $CD4^+CD6^+PD\text{-}1^{hi}$ and $CD8^+CD6^+PD\text{-}1^{lo}$ cells. Data are representative of 6 macaques. (B) The correlation of splenic $CD8^+CD6^+PD\text{-}1^+$ cells with the median SIV plasma viral load (VL) over weeks 12-40 post-infection. Data are from 25 macaques. (C) The correlation of $CD4^+CD6^+PD\text{-}1^{hi}$ cells with the median SIV plasma viral load (VL) over weeks 12-40 post-infection. Data are from 25 macaques. (D) The correlation of $CD4^+CD6^+PD\text{-}1^{lo}$ cells with the median SIV plasma viral load (VL) over weeks 12-40 post-infection. Data are from 25 macaques. For statistical analysis, non-parametric Spearman correlations were performed. All tests were two-tailed.
